# A Comparative Study of Chronic Subdural Hematoma in Patients With and Without Head Trauma: A Retrospective Cross Sectional Study

**DOI:** 10.3389/fneur.2020.588242

**Published:** 2020-11-27

**Authors:** Yunwei Ou, Xiaofan Yu, Xiaojuan Liu, Qian Jing, Baiyun Liu, Weiming Liu

**Affiliations:** ^1^Department of Neurosurgery, Beijing Tiantan Hospital, Capital Medical University, Beijing, China; ^2^Beijing Advanced Innovation Center for Big Data-Based Precision Medicine, Beihang University, Beijing, China; ^3^China National Clinical Research Center for Neurological Diseases, Beijing, China; ^4^Neurological Center, Ningxia People's Hospital, Ningxia, China

**Keywords:** clinical characteristic, common, chronic subdural hematoma, difference, head trauma

## Abstract

**Background:** The clinical features of chronic subdural hematomas (CSDHs) in patients with and without a history of head trauma have remained unclear. Here, we investigated differences in clinical characteristics in CSDH patients with and without head trauma.

**Methods:** We retrospectively collected clinical characteristics of CSDH patients who had undergone exhaustive drainage strategies via burr-hole craniostomies from August 2011 to May 2019. We divided patients into a trauma group (i.e., head trauma) and a no-trauma group. Chi-square tests or *t*-tests were used to analyze differences in clinical characteristics between the two groups. Multiple linear regression analysis was performed to analyze the relationships between the clinical characteristics and either reduction of the hematoma cavity or length of the hospital stay in CSDH patients with trauma.

**Results:** We collected data from 1,307 CSDH patients, among whom 805 patients had a history of head trauma whereas 502 patients did not. The mean age of patients with head trauma was 64.0 ± 16.1 years, while that of patients without head trauma was significantly younger at 61.5 ± 17.9 years (*p* = 0.010). Furthermore, more patients in the no-trauma group had a history of hypertension compared to those in the trauma group (40.2 vs. 32.9%, *p* = 0.007). Dizziness occurred in 29.2% of patients with trauma and in 23.1% of patients without trauma (*p* = 0.016). A greater number of patients with trauma showed a reduction of hematoma cavity after surgery compared to that of patients without trauma (*p* = 0.002). The length of hospital stay in patients with trauma was 7.9 ± 4.5 days, which was longer than that of patients without trauma (7.3 ± 3.7 days, *p* = 0.016). In contrast, there were no significant different differences between the two groups in terms of the densities of hematomas on computed-tomography scans, complications, mortality rates, recurrence rates, or outcomes.

**Conclusion:** Our findings indicate that there were some noteworthy differences in the clinical and pathogenic characteristics of CSDH patients with and without head trauma. However, our findings also indicate that if an optimal treatment method is employed, such as our exhaustive drainage strategy, similar treatment outcomes can be achieved between these groups.

## Introduction

Chronic subdural hematomas (CSDHs) generally increase with age and are more prevalent in older patients, especial in individuals >65 years old (1). Although many risk factors may contribute to the occurrence of CSDHs, the pathogenic mechanisms underlying CSDH have remained unclear. Head trauma is regarded as a primary cause of CSDH, and 30–75% of CSDH patients have a history of head trauma within 3 months prior to hospital admission (2, 3). However, some patients exhibit CSDH without any known cause. It has been reported that arachnoid cysts, brain surgery, coagulation factors, ventriculoperitoneal shunts, and hypertension may play roles in some spontaneous CSDH patients (4, 5). According to these differences, some reports have found that CSDH patients with a history of head trauma display different clinical and radiological characteristics, as well as different outcomes, compared to those of CSDH patients without a history of head trauma (6–8). However, these conclusions are based on a small number of cases, and these cases have exhibited high recurrence rates. Therefore, further studies with larger sample sizes are needed to more comprehensively analyze outcomes and clinical/radiological characteristics of CSDH patients with or without a history of head trauma in order to guide improved management and treatment of each type of CSDH patient.

In the present study, we retrospectively analyzed 1,307 CSDH patients, and we found that age, a history of hypertension, dizziness symptoms, and the interval from CSDH onset to admission were all significantly different between CSDH patients with and without head trauma. In contrast, there were no significant differences between the two groups in terms of radiological characteristics, outcomes, or recurrence. However, we found that CSDH patients with head trauma exhibited a larger reduction of hematoma cavity after surgery and a longer length of hospital stay compared with those of CSDH patients without a history of head trauma. Therefore, our finding may help to clarify the similarities and differences in the underlying characteristics of CSDH in patients with or without a history of head trauma.

## Methods

Symptomatic CSDH was defined as a predominantly subdural collection with hypodensity, isodensity, or mixed density in computed tomography (CT) scans; and patients with other causes of CSDH identified during operation or subsequent treatment (e.g., empyema, arachnoid cyst) were excluded from further analysis in the present study, and patients taking steroids or atorvastatin for CSDH before operation were also excluded. Following these exclusions, data from 1,307 patients with a primary or recurrent symptomatic CSDH confirmed on cranial imaging were collected from August 2011 to May 2019 at the Department of Neurosurgery in Beijing Tiantan Hospital. Age was not used as an inclusion or exclusion criterion. According to our previous report, an exhaustive drainage strategy via burr-hole craniostomy was carried out in all patients (3). Clinical parameters—such as age, gender, medical history, and history of head trauma events—were collected from each patient. Complications after operations were also recorded, including pneumonia, pulmonary embolism, heart failure, heart attack, stroke, fever, and seizure. A head trauma event was defined as a history of head trauma within 3 months prior to hospital admission. At sixth months after patients were discharged, their CT scans were examined and their corresponding modified Rankin scale (MRS) scores were used by two independent neurosurgeons (via phone correspondence) to analyze each patient's outcome. An MRS score of 0–3 indicated a good outcome, while a score of 4–6 indicated an inferior outcome. The Bender grade was used to analyze the preoperative statuses of patients (9), while a CT grading system by Stanisic et al. was utilized to classify the density of each hematoma (10). The postoperative CSDH recurrence rate was defined as the rate of reoperation to treat recurrent ipsilateral hematoma within 6 months after the original operation. Preoperative and postoperative hematoma volumes were calculated by the Coniglobus formula. Informed consent from patients and ethics approval from the Institutional Research Ethics Committee were obtained (NO. KY 2020-094-02).

### Statistical Analyses

For all clinical parameters, means± standard deviations were used for continuous variables, while numbers of patients and percentages were used for categorical variables. Measurement data were tested for normality before statistical analyses, and non- normally distributed data were analyzed via non-parametric tests. Chi-square tests or *t*-tests were used to analyze differences in clinical characteristics in CSDH patients with and without head trauma. Multiple linear regression analysis was performed to analyze relationships between clinical characteristics and either reduction of the hematoma cavity or length of the hospital stay in CSDH patients with head trauma. Statistical analyses were performed by SPSS software version 17.0.0, and a *p*-value < 0.05 was considered significant.

## Results

### Relationships Between Clinical Characteristics and Head Trauma

To analyze differences in baseline characteristics of CSDH patients with or without head trauma, we divided patients into a trauma group (i.e., head trauma) and no-trauma group. Among all CSDH patients, 805 cases had a history of head trauma from which they had each since recovered. As shown in Table 1, the male:female ratio was 4.6:1.0 (661:144 patients) in patients with head trauma, and 5.0:1.0 (419:83 patients) in patients without head trauma (*p* = 0.530). The mean age of patients with head trauma was 64.0 ± 16.1 years, while that of patients without head trauma was significantly younger at 61.5 ± 17.9 years (*p* = 0.010). In contrast, we did not find any significant differences in terms of a history of smoking, drinking, diabetes, cardiac disease, brain infarction, or antithrombosis between CSDH patients with and without head trauma. However, more patients exhibited hypertension in the no-head trauma group than in the trauma group (40.2 vs. 32.9%, *p* = 0.007).

**Table 1:** Relationship between clinical characteristics and trauma in patients with chronic subdural hematoma.

**Characteristics analyzed**	**Trauma (*****n*****)**		***p***
	**Yes (805)**	**No (502)**	
Gender (Male: Female)	661:144	419:83	0.530
Age	64.0 ± 16.1	61.5 ± 17.9	0.010
Personal/Past history			<0.001
Smoking	216 (26.8)	133 (26.5)	0.893
Drinking	155 (19.3)	94 (18.7)	0.813
Hypertension	265 (32.9)	202 (40.2)	0.007
Diabetes	160 (19.9)	97 (19.3)	0.807
Cardiac diseases	29 (3.6)	20 (4.0)	0.724
Brain infarction	93 (11.6)	50 (10.0)	0.370
History of antithrombotic	92 (11.4)	52 (10.4)	0.548
AC/V-P shunt *n* (%)	68 (8.4)	47 (9.4)	0.570
**Symptoms**			
Headache *n* (%)	479 (59.5)	279 (55.6)	0.162
Dizziness *n* (%)	235 (29.2)	116 (23.1)	0.016
Limb weakness *n* (%)	457 (56.8)	260 (51.8)	0.079
Dysphasia *n* (%)	80 (9.9)	42 (8.4)	0.342
Disturbance of consciousness *n* (%)	35 (4.3)	25 (5.0)	0.595
Interval from onset to admission	8.5 ± 6.3	11.5 ± 19.3	0.001
Bender grades			<0.001
0	31 (3.9)	35 (7.0)	
I	307 (38.1)	249 (49.6)	
II	431 (53.5)	198 (39.4)	
III	36 (4.5)	20 (4.0)	
Unilateral/bilateral hematoma			0.068
Left	314 (39.0)	227 (45.2)	
Right	266 (33.0)	156 (31.1)	
Bilateral	225 (28.0)	119 (23.7)	
Types of hematoma			0.461
A	108 (13.4)	71 (14.1)	
B	234 (29.1)	148 (29.5)	
C	177 (22.0)	88 (17.5)	
D	65 (8.1)	48 (9.6)	
E	33 (4.1)	26 (5.2)	
F	34 (4.2)	27 (5.4)	
G	154 (19.1)	94 (18.7)	
Preoperative volume (ML)	99.6 ± 28.9	99.2 ± 31.5	0.805

Headache was the most common symptom in each of the two groups (59.5, 55.6%, respectively), whereas a disturbance of consciousness was the least common symptom in each group (4.3, 5.0%, respectively). We found that dizziness occurred in 29.2% (235/805) of patients with head trauma, which was significantly higher than the 23.1% (116/502) of patients without head trauma that experienced dizziness (*p* = 0.016). In contrast, no significant differences were found between the two groups in terms of other symptoms, such as limb weakness or dysphasia. Furthermore, according to Bender grades, more CSDH patients with head trauma exhibited a Bender grade II while more CSDH patients without head trauma exhibited a Bender grade I (*p* < 0.001), suggesting that patients with head trauma suffered from more severe conditions. The average interval from CSDH onset to admission was 8.5 ± 6.3 days in patients with head trauma, which was significantly shorter than the 11.5 ± 19.3 days in patients without head trauma (*p* = 0.001).

The hematomas in CSDH patients displayed different densities on CT scans, in terms of hypodensities, isodensities, or mixed densities. We further analyzed differences in hematoma densities according to the CT grading system of Stanisic et al. (10), and found that they were not significantly different between patients with and without head trauma. Furthermore, there was no significant difference in the anatomical side at which the hematomas were located in either of the two groups. Finally, the average preoperative volume of hematomas was 99.6 ± 28.9 mL in the trauma group, and 99.2 ± 31.5 mL in the no-trauma group (*p* = 0.805).

### Relationships Between Clinical Characteristics and Head Trauma After Surgery

According to our previous report (3), an exhaustive drainage strategy via burr-hole craniostomy was carried out foreach CSDH patient after they were admitted to our hospital. As shown in Table 2, the reduction of hematoma cavity after surgery was 58.9 ± 21.4% in the head trauma group, and 55.2 ± 22.2% in the no-trauma group (*p* = 0.002), suggesting that brain plasticity in CSDH patients without head trauma may have been worse than that of patients with head trauma. After the operation, the length of hospital stay in patients with head trauma was 7.9 ± 4.5 days, which was longer than that of patients without trauma (7.3 ± 3.7 days, *p* = 0.016).

**Table 2:** Relationship between clinical characteristics and trauma after surgery in CSDH patients.

**Characteristics analyzed**	**Trauma** ***n*** **(%)**		***p***
	**Yes (805)**	**No (502)**	
Reduction of hematoma cavity (%)	58.9 ± 21.4	55.2 ± 22.2	0.002
Symptom disappeared or alleviated (day)	2.5 ± 1.9	2.7 ± 2.2	0.162
Duration of drainage catheter (day)	3.5 ± 1.8	3.4 ± 2.1	0.651
Length of hospital stay (days)	7.9 ± 4.5	7.3 ± 3.7	0.016
Use of urokinase	434 (53.9)	271 (54.0)	0.980
Frequency of urokinase used	1.7 ± 0.9	1.6 ± 0.9	0.678
Recurrence requiring reoperation	13 (1.6)	11 (2.2)	0.183
Complications	55 (6.8)	32 (6.4)	0.747
Death (hospital stay)	4 (0.5)	4 (0.8)	0.490
Death (in 6 month)	12 (1.5)	6 (1.2)	0.656
Outcome (MRS)			0.233
0–3	778 (96.6)	488 (97.2)	
4–6	19 (2.4)	13 (2.6)	

During our exhaustive drainage strategy, each hematoma was drained by the catheter, which was then removed when drainage ceased. We found that the duration of the drainage catheter in the trauma group was 3.5 ± 1.8 days, while it was 3.4 ± 2.1 days in patients without head trauma (*p* = 0.651). Otherwise, urokinase was used to promote hematoma drainage during execution of our strategy. There were no differences in the use of urokinase or the frequency of urokinase use between patients with and without head trauma.

A low recurrence requiring reoperation is one of the most important issues to consider before choosing a suitable treatment for patients with CSDH. We found that the recurrence requiring reoperation in patients with head trauma was 1.6% (13/805), and was 2.2% (11/502) in patients with head trauma. Interestingly, we did not find a significant difference between the two groups in terms of this recurrence rate. Additionally, patients with CSDH can experience complications after operation, such as pneumonia, pulmonary embolism, heart failure, heart attack, stroke, fever, and/or seizure. We found that 6.8% (55/805) of CSDH patients with head trauma exhibited complications, and 6.4% (32/502) of CSDH patients without head trauma exhibited complications. We did not identify any significant differences between the two groups in terms of post-operation complications. Furthermore, no significant differences were found between on the two groups in terms of mortality rates during their hospital stays or at sixth months after being discharged. The outcomes in patients with head trauma were excellent, as 96.6% of these patients obtained MRS scores of 0–3. Meanwhile, patients without head trauma also had favorable outcomes, such that 97.2% of them obtained MRS scores of 0–3, revealing that there was no significant difference between the two groups based on this parameter.

According to the above analyses, reduction of hematoma cavity after surgery in CSDH patients without head trauma was less than that of CSDH patients with head trauma, and we further analyzed which clinical parameters contributed to this differential phenotype via multiple linear regression analyses. As shown in Table 3, among CSDH patients with trauma, cases with an AC/V-P shunt (B = 0.080, Beta = 0.103, *p* = 0.003) or unilateral hematoma (B = 0.068, Beta = 0.143, *p* < 0.001) were correlated with a greater reduction of hematoma cavity after surgery. Interestingly, patients with an AC/V-P shunt or unilateral hematoma were also significantly younger (*p* < 0.01, respectively). Patients with a long interval from CSDH onset to admission (B = −0.005, Beta = −0.133, *p* < 0.001), long duration of their drainage catheter (B = −0.011, Beta = −0.090, *p* = 0.009), or a history of cardiac disease (B = −0.085, Beta = −0.074, *p* = 0.031) were correlated with less reduction of the hematoma cavity after surgery. Patients with cardiac disease were older than those without cardiac disease (*p* < 0.001), while a long duration of their drainage catheter was also more common in older patients (*p* = 0.014).

**Table 3:** Multiple linear regression analyses of characteristics related to reduction of hematoma cavity after surgery in CSDH patients with trauma.

**Characteristics analyzed**	**B**	**Beta**	**P**
AC/V-P shunt	0.080	0.103	0.003
Interval from onset to admission	−0.005	−0.133	<0.001
Cardiac diseases	−0.085	−0.074	0.031
Duration of drainage catheter	−0.011	−0.090	0.009
Unilateral/bilateral hematoma	0.068	0.143	<0.001

Finally, we further analyzed the influencing factors of the length of hospital stay in patients with head trauma (Table 4). We found that patients with brain infarction (B = 1.591, Beta = 0.114, *p* < 0.001) or a longer duration of their drainage catheter (B = 0.709, Beta = 0.287, *p* < 0.001) had a longer length of hospital stay, and complications in patients were also correlated with a longer length of hospital stay (B = 5.764, Beta = 0.326, *p* < 0.001).

**Table 4:** Multiple linear regression analyses of characteristics related to length of hospital stay after surgery in CSDH patients with trauma.

**Characteristics analyzed**	**B**	**Beta**	**P**
Brain infarction	1.591	0.114	<0.001
Duration of drainage catheter	0.709	0.287	<0.001
Complications	5.764	0.326	<0.001

## Discussion

Few previous studies have investigated similarities and differences in characteristics of CSDH patients with or without head trauma, and the results of these studies have remained controversial due to their low sample sizes. Here, we analyzed the clinical characteristics of 1,307 CSDH patients with or without head trauma, which currently represents the largest sample size of any such study. Although CSDH can be identified via an abnormal subdural collection of liquefied blood, the pathogenic mechanism of CSDH has remained unclear. At present, one of the most commonly posited mechanisms of CSDH is that it derives from bridging veins, cortical arteries, and/or cortical veins tearing after mild head injury with subsequent bleeding and induction of hematoma (11). Therefore, head trauma has been considered as one of the most common risk factors for the occurrence of CSDH. To identify differential clinical characteristics, recurrence rates, and outcomes of CSDH patients with and without head trauma, we divided 1,307 CSDH patients into a trauma group (i.e., head trauma) and no-trauma group. We found that 61.6% of our CSDH patients has a history of head trauma. Furthermore, we found that older patients were more likely to have a history of head trauma, which is not consistent with the findings of several previous reports (6, 12, 13). This disparity between studies may be due to some elderly patients not noticing or remembering that they may have had a history of mild head trauma in these previous studies. Additionally, we found that the reduction of hematoma cavity after surgery in CSDH patients with head trauma was greater than that in CSDH patients without head trauma. Among CSDH patients with head trauma, those with an AC/V-P shunt or unilateral hematoma had a greater reduction of the hematoma cavity and were younger; patients with a long duration of their drainage catheter or a history of cardiac disease exhibited less of a reduction of the hematoma cavity, and these patients were also more likely to be older patients. All of these results suggest that age is an important factor in the reduction of hematoma cavity in patients with head trauma. This phenomenon may be due to age-related brain atrophy, such that the brain gradually loses its plasticity and does not show good re-expansion after removal of a hematoma.

According to our previous reports and other studies (3, 7), only 50–70% of CSDH patients have a history of head trauma, suggesting that other risk factors may also contribute to CSDH. Indeed, in addition to head trauma, many other risk factors have been identified in the process of CSDH, such as alcoholism, coagulopathies, and cerebrospinal fluid shunts (5, 14, 15). In the no-trauma group in our present study, we found that 40.2% of patients had a history of hypertension, which was significantly different compared with that of the trauma group. This result suggests that hypertension may play an important role in the process of CSDH in patients without head trauma. The reason for this phenomenon may be attributed to a fluctuation in blood pressure, which makes bridging veins, cortical arteries, and/or cortical veins more easily torn, suggesting that controlling hypertension may be important for the prevention of CSDH.

Headache is the most common symptom in patients with CSDH (3). However, patients with or without head trauma may exhibit differential symptoms. It has been reported that patients without head trauma have a higher rate of muscle weakness (6). However, we did not find any difference in muscle weakness between the two groups in our present study and found that patients with head trauma displayed a higher rate of dizziness. Furthermore, we did not find any other differences between the two groups in terms of any other symptoms. Moreover, according to the Bender grade system, we found that patients with head trauma exhibited more severe conditions, which is consistent with a previous report (6).

Hematoma densities on CT scans display different densities, such as homogeneous, separated, mixed-density, and high-density features. It has been reported that hematoma density is correlated with different clinical characteristics, and patients with high-density CT areas show the largest incidence of recurrence (16, 17). Jun et al. found that homogeneous density mainly occurred in patients with head trauma, while mixed density was mostly found in patients without head trauma (6). In our present study, we classified hematomas into seven subtypes according to the different densities of hematomas on CT scans (10) and found that there were no significant differences between CSDH patients with and without head trauma.

Complications, mortality rates, recurrence rates, and outcomes are important indexes for evaluating effects of different kinds of treatments. It has been shown that CSDH patients without head trauma exhibit a higher mortality rate than that of CSDH patients with head trauma (6). However, in our present report, we found that 0.5% of CSDH patients with head trauma died during their hospital stay, whereas 0.8% of CSDH patients without head trauma died during their hospital stay. Furthermore, 1.5% of patients died during follow-up in the trauma group, while 1.2% of patients died during follow-up in the no-trauma group. None of these results were significantly different between the two groups, suggesting that mortality rates over short-term or long-term periods are not different in CSDH patients with or without trauma. A history of head trauma is correlated with a poor outcome at long-term follow-ups in CSDH patients (8). However, Jun et al. found that CSDH patients without head trauma had poor outcomes (6). In our present study, the length of the hospital stay in patients with head trauma was longer than that of patients without head trauma, and further analyses revealed that brain infarction, duration of drainage catheter, and complications were correlated with the length of the hospital stay. Furthermore, complication was the most significant factor among these parameters, suggesting that a longer length of hospital stay may cause a poor outcome or more complications. However, we found that there were no significant differences between the two groups in terms of complications or outcomes. The reason for this lack of any differences between groups may be due to the fact that we performed an exhaustive drainage strategy via burr-hole craniostomy in all patients in the present study (3), which may have helped to maximally reduce hematomas, minimize recurrence rates, and yield favorable outcomes. Finally, our present study had some limitations in terms of it being a retrospective single-center study with a relatively small sample size. Therefore, in the future, we plan to conduct a multi-center prospective study and/or randomized-controlled trial to verify or refute our present results.

## Conclusions

In conclusion, hypertension may be a risk factor in CSDH patients without head trauma. In our present study, we found that there were no significant differences in terms of densities of hematomas on CT scans, complications, mortality rates, recurrence rates, or outcomes between CSDH patients with and without head trauma. Taken together, our findings suggest that if an optimal treatment method is employed, such as our exhaustive drainage strategy, similar treatment outcomes can be achieved between these groups.

## Data Availability Statement

The raw data supporting the conclusions of this article will be made available by the authors, without undue reservation.

## Ethics Statement

The studies involving human participants were reviewed and approved by Beijing Tiantan Hospital, Capital Medical University, China. Written informed consent to participate in this study was provided by the participants' legal guardian/next of kin.

## Author Contributions

YO and WL designed the research. XY, XL, and QJ collected data. YO drafted the manuscript and takes the responsibility for the integrity of the data and the accuracy of the data analysis. BL supervised the project. All authors contributed to the article and approved the submitted version.

## Conflict of Interest

The authors declare that the research was conducted in the absence of any commercial or financial relationships that could be construed as a potential conflict of interest.
